# An Overview of Zeolites: From Historical Background to Diverse Applications

**DOI:** 10.3390/molecules30204036

**Published:** 2025-10-10

**Authors:** Abdulrahman Masoud Alotaibi

**Affiliations:** Department of Physics, Faculty of Science, Umm Al-Qura University, Makkah 21955, Saudi Arabia; amaotabey@uqu.edu.sa

**Keywords:** natural zeolites, radioactive waste remediation, wastewater treatment, agriculture, aquaculture, construction, carbon capture, sustainable materials

## Abstract

This article provides a concise review of zeolites, encompassing their definition, historical background, natural occurrence, geographical distribution, and diverse applications. As a versatile and cost-effective microporous material, zeolites have demonstrated significant potential across various fields, including wastewater treatment, radioactive waste management, agriculture, aquaculture, construction, medicine, and biotechnology. The review also highlights current challenges and explores emerging opportunities for future applications of zeolites. While this brief overview does not fully capture the complexity of the subject, it aims to spark interest in further research into this promising material.

## 1. Definition and History

Natural zeolites are crystalline aluminosilicates distinguished by a microporous, three-dimensional structure formed by interconnecting tetrahedral units of silica (SiO_4_) and alumina (AlO_4_), linked by shared oxygen atoms. This distinctive structural arrangement produces a porous network with linked cavities and channels, which generally accommodate water molecules and exchangeable cations, including potassium (K^+^), sodium (Na^+^), calcium (Ca^2+^), and magnesium (Mg^2+^). The cations are essential for neutralizing the aluminosilicate framework negative charge, thus enhancing the ion-exchange and adsorption characteristics that make zeolites highly advantageous in diverse environmental and industrial applications [1,2].

Axel Fredrik Cronstedt, a Swedish mineralogist, discovered the first zeolite mineral, stilbite, in 1756. He noted the emission of steam from the mineral stilbite when subjecting it to high temperatures; the steam was generated from water that was previously adsorbed within the mineral’s structure. Based on this observation, he named the material “zeolite”. The term zeolite originates from two Greek words: zeo (to boil) and lithos (stone), referring to the mineral’s apparent “boiling” behavior when heated [2].

Since the discovery of natural zeolite, it has been used in many applications, for example, as pozzolans and lightweight aggregates in concretes and cements, as adsorbents in the take-up of cesium and strontium from nuclear waste and in the removal of ammonia. Because of the low production of natural zeolite and the failure to meet demands, the business of synthetic zeolite has become popular. However, this business was put on hold in the late 1950s, due to the discovery of large beds of zeolite-rich sediments which are containing around 95% natural zeolite mineral. The formation of these sediments occurred via the process of volcanic glass alteration in both marine and lake waters. The first zeolite sediments were discovered in America. The zeolite beds were simply mined and processed for diverse applications [3].

**Figure 1:**
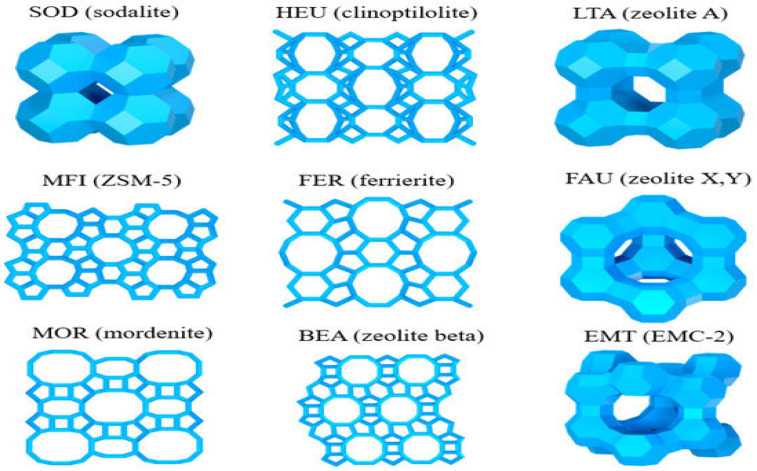
The structure of zeolites.

Since Cronstedt’s discovery of the first natural zeolite in 1756, the development of this material has advanced significantly. Early studies focused mainly on naturally occurring minerals such as stilbite, clinoptilolite, chabazite, and mordenite, which were widely utilized in agriculture and industry [3]. However, the limited supply of natural zeolites led to the rapid growth of synthetic zeolite research and production in the mid-20th century. Synthetic zeolites are obtained under controlled hydrothermal conditions, allowing scientists to tailor their pore size, framework composition, and physicochemical properties for specific industrial applications [4,5]. To date, more than 200 unique zeolite framework structures have been reported, with approximately 40 occurring naturally, while the remainder are synthesized in laboratories [6]. Representative examples include the naturally abundant clinoptilolite and chabazite, alongside widely applied synthetic types such as zeolite A, X, and Y [5,6,7]. Figure 1 illustrates the structure of zeolites, providing a clearer picture of their framework diversity [8]. This historical transition from natural discovery to synthetic innovation highlights the critical role of zeolite development in shaping its versatile applications across science and technology.

In addition to zeolites, the past two decades have witnessed the rapid rise in other porous materials, particularly metal–organic frameworks (MOFs), which have attracted significant research interest due to their exceptionally high surface areas and structural tunability (e.g., modular metal nodes and organic linkers). These features allow MOFs to achieve gas uptake capacities and specificities often exceeding those of zeolites [9,10]. However, their commercial adoption remains limited, primarily because of challenges in stability under moisture, synthetic cost, and scalability [11]. In contrast, zeolites have already established themselves as one of the most commercially successful classes of porous materials, thanks to their superior thermal and chemical stability, cost-effective synthesis routes, availability of natural deposits, and decades of industrial utilization in catalysis, gas separation, detergents, and water treatment [7,12,13]. This comparative perspective underscores the continuing industrial dominance of zeolites, while situating them within the broader landscape of emerging porous materials.

## 2. Occurrence and Geographical Distribution of Natural Zeolites

**Figure 2:**
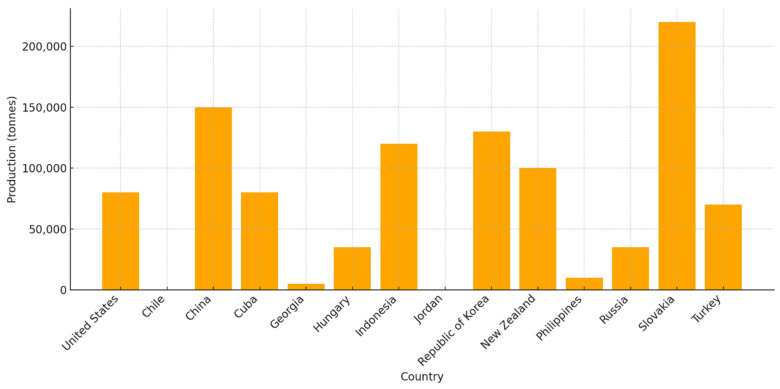
Main producers of natural zeolite.

Since the initial discovery of natural zeolites within volcanogenic sedimentary rocks, numerous types of natural zeolites have been discovered throughout various global locations. In the last decade, natural zeolites have garnered significant attention for their diverse applications in fields such as catalysis, adsorption, the construction industry, energy systems, and environmental remediation. This growing interest is primarily attributed to their distinctive physicochemical properties. The world’s natural zeolite consumption was estimated in 1988 to be 3.98 million tonnes, and it was projected to reach 5.5 million tonnes by 2010 [1,14]. According to the U.S. Geological Survey in 2025, the annual world production of natural zeolite is approximately 1,000,000 tonnes. Main producers in 2025 included Slovakia (220,000 tonnes), China (150,000 tonnes), the Republic of Korea (130,000 tonnes), Indonesia (120,000 tonnes), New Zealand (100,000 tonnes) and the United States (81,000 tonnes), as presented in Figure 2.

Natural zeolites occur in a diverse array of rocks, spanning varied ages and geological conditions. Traditionally, geologists have posited that zeolites are generated by the interaction of water with rocks across various geological settings. Numerous instances of zeolites have been documented as accessory minerals in basic igneous rocks and inside the vugs and cavities of basalts, as well as being primary components of several bedded pyroclastic deposits. Natural zeolites are acknowledged as the most prevalent and extensive authigenic silicates in volcanic sedimentary rocks. The majority of natural zeolites are generated during the diagenetic process within sedimentary rocks. The predominant natural zeolites found in sedimentary contexts are clinoptilolite, chabazite, heulandite, analcime, mordenite, phillipsite, erionite, and laumontite, with clinoptilolite being the most prevalent. The mineralogical, physical, and chemical characteristics of natural zeolites are contingent upon their geological formations [15,16].

Natural zeolites in sedimentary rocks have been classified according to their hydrologic or geologic development processes into seven distinct categories: (1) volcanic and non-volcanic sediments in alkaline soils; (2) volcanic sediments in closed hydrologic system deposits (alkaline, saline lake deposits); (3) volcanic sediments in open hydrologic system deposits (groundwater or freshwater lake environments); (4) volcanic and non-volcanic sediments in marine deposits (both near-shore and deep-sea environments); (5) volcanic sediments in burial diagenetic or burial metamorphic deposits; (6) non-volcanic materials transformed in various aqueous environments; and (7) volcanic materials in hydrothermal or hot spring deposits [15].

## 3. Structure of Zeolite

Zeolites are microporous crystalline aluminosilicates composed of SiO_4_ and AlO_4_ tetrahedra, where each central silicon or aluminum atom is coordinated with four oxygen atoms. These tetrahedra, known as primary building units (PBUs), connect via shared oxygen atoms to form secondary building units (SBUs), which further assemble into the zeolite’s three-dimensional framework structure [4,16]. The framework contains interconnected channels and cages—micropores—that host exchangeable extra-framework cations (e.g., Na^+^, K^+^, Ca^2+^, Mg^2+^) and water molecules. The substitution of Si^4+^ by Al^3+^ introduces a net negative charge, balanced by these mobile cations, endowing zeolites with cation exchange capacity [7,16,17,18]. SBUs exist in various geometric forms (e.g., rings, polyhedra), with up to 16 tetrahedral atoms (T = Si or Al), forming a variety of pore architectures. To date, 23 distinct SBU types have been identified [6]. The most common formula of natural zeolites is:

M_2/n_O.Al_2_O_3_.xSiO_2_.yH_2_O

where M is any alkali and earth alkaline atom, n is a charge on the atom, x is the number of Si tetrahedra in the structure, which varies from two to ten, and y is the number of water molecules in the natural zeolite, which varies from 2 to 7 [19].

## 4. Characterization Techniques

Characterization techniques that provide insights into their structural, textural, and chemical features. X-ray diffraction (XRD) is the primary tool for determining crystalline phases and framework types, confirming zeolite identity and purity [4,6]. Fourier transform infrared spectroscopy (FTIR) is widely applied to probe framework vibrations, hydroxyl groups, and Brønsted or Lewis acid sites, which are central to catalytic performance [7,20].

The porosity and surface area of zeolites are typically examined by nitrogen adsorption–desorption isotherms (BET analysis), which quantify micropore volume and specific surface area, parameters crucial for adsorption and gas separation applications [12]. Scanning electron microscopy (SEM) and transmission electron microscopy (TEM) reveal crystal morphology, particle size, and surface defects, while energy-dispersive X-ray spectroscopy (EDS) coupled with SEM provides elemental composition [16,20]. Additionally, solid-state nuclear magnetic resonance (NMR), particularly ^27^Al and ^29^Si MAS-NMR, yields information on the coordination environment of framework atoms, which governs acidity, ion-exchange capacity, and framework stability [7]. Thermogravimetric analysis (TGA) and differential scanning calorimetry (DSC) are commonly used to evaluate thermal stability and water content, both of which strongly influence zeolite suitability in gas dehydration, catalysis, and construction applications [2,12].

Importantly, these characterization techniques not only describe zeolite structures but also provide evidence that substantiates their applications. XRD confirms framework stability and phase purity, ensuring zeolites employed in catalysis and adsorption exhibit the required crystallinity. SEM/TEM directly links particle size and morphology to diffusion pathways and adsorption efficiency. FTIR detects hydroxyl groups and acid sites, establishing the connection between framework vibrations and catalytic or adsorption activity. Solid-state NMR validates the presence and distribution of Brønsted and Lewis acid sites, clarifying their role in ion exchange and catalysis. Adsorption isotherms (BET) confirm microporosity and surface area, directly validating zeolite suitability for gas separation, purification, and storage. Complementary spectroscopic methods such as UV-Vis and Raman further link framework integrity and active site distribution to reactivity and stability.

In addition to these conventional methods, several emerging and less commonly employed characterization techniques are gaining attention for their ability to provide deeper mechanistic insights. In situ and operando spectroscopy (e.g., FTIR, Raman, UV-Vis) allow real-time monitoring of zeolite structural changes and active sites under working conditions, thereby bridging the gap between static characterization and catalytic performance [7,12,21]. Synchrotron-based methods, including X-ray absorption spectroscopy (XAS), near-ambient-pressure XPS, small-angle X-ray scattering (SAXS), and operando diffraction, offer unparalleled resolution and sensitivity, enabling the identification of framework distortions, active metal species, and dynamic processes at the atomic scale [13,22]. Moreover, advanced solid-state NMR and EPR techniques, such as high-field and multidimensional NMR, dynamic nuclear polarization (DNP-NMR), and pulsed EPR, provide detailed information on framework environments, paramagnetic centers, host–guest interactions, and catalytic intermediates [7,23,24].

Although these techniques have not yet been widely adopted in routine zeolite studies due to cost, instrumental accessibility, or complexity, they represent powerful tools for establishing more direct links between structure and function. Highlighting these opportunities suggests promising directions for future research, particularly in developing a more fundamental understanding of zeolite-based applications [13,21,23].

## 5. Zeolite Synthesis and Production Strategies

While naturally occurring zeolites have been widely utilized, the increasing demand for specific structural and functional properties has driven extensive research into synthetic zeolites. Laboratory synthesis is typically achieved under hydrothermal conditions, where silica and alumina sources crystallize in alkaline aqueous solutions at controlled temperatures and pressures [4,5]. This method allows for precise control over framework topology, pore size, and chemical composition. As a result, more than 160 synthetic zeolite frameworks have been developed, complementing the ~40 naturally occurring types [6].

Commercial production of synthetic zeolites has become a major industry, with zeolite A, X, Y, and ZSM-5 being among the most widely produced due to their applications in catalysis, adsorption, and ion-exchange. Beyond hydrothermal synthesis, recent strategies include microwave-assisted synthesis, templating methods, and green synthesis approaches, which aim to reduce energy consumption and improve sustainability [5,13]. These innovations reflect the critical importance of synthetic zeolite production in meeting the growing industrial and environmental demands.

## 6. Applications of Zeolites

The wide range of applications of zeolites is directly related to their intrinsic physicochemical properties. Their high ion-exchange capacity, arising from the negatively charged aluminosilicate framework, underpins many environmental applications such as wastewater treatment, agriculture, aquaculture, and radioactive waste remediation, where selective removal of metal ions, ammonium, and radionuclides is required. The shape-selective microporosity of zeolites enables them to function as molecular sieves, forming the basis for gas dehydration, air separation, and purification processes, as well as for pollutant adsorption in aqueous systems.

Moreover, the tunable acidity of zeolites, which can be controlled by adjusting the Si/Al ratio or through ion-exchange modifications, provides the catalytic activity necessary for chemical transformations and sustainable industrial processes. Their high thermal and chemical stability ensures long-term performance in demanding environments, supporting applications in construction materials and nuclear waste remediation. Finally, the biocompatibility and adsorption capacity of zeolites allow their use in medical and biotechnological fields, including detoxification, wound healing, and controlled drug delivery.

By explicitly establishing these property–application relationships, the scientific rationale behind zeolite applications becomes clearer. This framework highlights how fundamental features such as stability, porosity, acidity, and ion-exchange capacity directly translate into practical performance across environmental, industrial, medical, and emerging technological fields.

**Figure 3:**
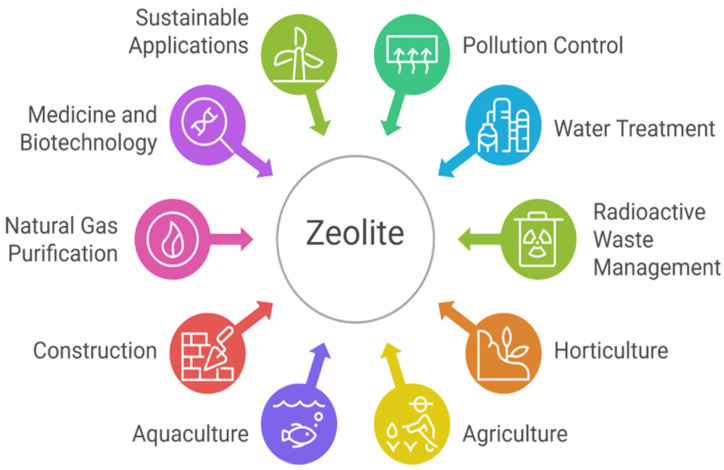
Zeolite applications.

Zeolites have attracted significant interest from researchers and scientific societies throughout the years owing to their adaptability and versatility. Since their original discovery in 1756 by the Swedish mineralogist, natural zeolites have been recognized as effective molecular sieves, ion exchangers, catalysts, and adsorbents [5,20]. Due to their distinctive properties, zeolites have been extensively employed in various applications, including pollution control, water treatment, radioactive waste management, horticulture, agriculture, aquaculture, the construction sector, natural gas purification, and other diverse uses [3]. Figure 3 illustrates a visual summary of the major applications of zeolite across different industries and environmental sectors.

In addition to its conventional uses, zeolite is assuming an increasingly important role in many sustainable applications, particularly in environmental enhancement and renewable energy sectors. For example, fuel cells, thermal energy storage, and biomass conversion [12].

Extensive studies have been conducted on zeolite, with estimates suggesting that 10% of natural zeolites are allocated for wastewater treatment, nuclear waste management, horticulture, odor control, animal feed, and various other applications, while the remaining 90% are utilized within the construction industry [25,26]. The following section provides a general overview of the various applications of zeolites.

### 6.1. Wastewater Treatment

The earliest usage of natural zeolites was in 1777 by Scheele and Fontana as adsorbents. Since then, their adsorption properties have allowed them to be utilized in a variety of operations that are used to solve environmental issues. Subsequently, natural zeolites have been found to be promising adsorbents for many molecules such as NH_3_, H_2_O, SO_2_, H_2_S, CO_2_, NO and NO_2_, to mention a few. Increasing demands for a higher quality drinking water have led to a global need to purify waters from different sources including industrial, natural, municipal and agricultural wastewaters. Consequently, the utilization of natural zeolites in the removal of contaminants from wastewater has gained considerable interest, culminating in extensive studies [16,20].

Many industrial processes, such as manufacturing and mining, generate wastewater streams that have different physical and chemical characteristics. They may contain a number of metal ions like Pb, Sb, Cu, Co, Zn, Ni, and Cr, together with other waste liquids which are produced by the mineral processing or metal finishing industries. These metals are toxic even at very low concentrations, and may exist in these waste waters at high concentrations. The presence of these metal ions in wastewaters is a serious threat to the environment and the health of human beings [16,20,27].

A lot of investigations have been devoted to the study of wastewater treatment and water purification methods. Case studies were carried out on these subjects include the removal of heavy metals Pb (II), Cd (II) and Cu (II) from industrial wastewater using natural zeolites was studied, this study indicated the suitability of natural zeolites for the removal of Pb^2+^, Cd^2+^ and Cu^2+^ ions [28]; the adsorption capacity of natural zeolite (clinoptilolite) and possibility for removing pesticides from waters was investigated, it has been found that clinoptilolite has the potential to be utilized to remove pesticides that present in surface water and groundwaters [29]; and the removal of heavy metals from acid mine drainage generated from the area of copper mine using natural zeolite was tested, the results confirmed that natural zeolite can be utilized as an efficient adsorbent for copper ions [30].

The presence of ammonia (NH_4_^+^) in water may lead to the growth of micro-organisms and the appearance of taste and odor in water. The presence of (NH_4_^+^) in water also makes it dangerous for human consumption. The maximum allowed concentration of (NH_4_^+^) in water is 0.5 mg/L. Therefore, it is essential to remove (NH_4_^+^) from water before it can be utilized for drinking. Zeolite is one of the most commonly used materials for removing ammonia ions [31]. For example, chitosan, zeolite, activated carbon and bleached fibre have been investigated for the removal of ammonium ions by adsorption; in this investigation zeolite showed the highest adsorption capacity for ammonium ions compared with the other materials [32]. The efficiency of ammonia removal using clinoptilolite was studied, and based on the results, clinoptilolite can be effectively applied in the treatment of wastewater, for both economic and technical aspects [33].

Collectively, these findings suggest that the application of natural zeolites in wastewater treatment holds significant promise due to their excellent adsorption capabilities for a wide range of contaminants, including heavy metals, pesticides, and ammonium ions. Their natural abundance, cost-effectiveness, and environmental friendliness make them especially suitable for treating industrial, agricultural, and municipal wastewater. Numerous studies confirm their effectiveness in removing hazardous substances such as Pb^2+^, Cd^2+^, Cu^2+^, and NH_4_^+^, thereby contributing to safer water resources and improved public health. As water quality becomes an increasingly critical global concern, natural zeolites continue to emerge as a sustainable and efficient solution to modern wastewater treatment challenges.

### 6.2. Agriculture

Agronomy and Horticulture. Natural zeolites have many positive impacts on the properties of soil, such as raising the cation exchange capacity and moisture of soil, increasing crop yields, decreasing plant uptake of contaminants and enhancing hydraulic conductivity. Natural zeolites are extensively used as slow-release fertilizers and soil amendments [34]. The Agricultural Improvement Section of the Yamagata Prefectural Government in Japan observed significant improvements in crop yields when they used clinoptilolite-rich tuff as a conditioner for the soil. Adding zeolite at a rate of approximately 4 to 8 tons per acre resulted in increased eggplant yields by 19 to 55%, apple yields by 13 to 38%, wheat yields by 13 to 15%, and carrot yields by 63% [3]. The addition of clinoptilolite resulted in increased crop yields of wheat, barley, potatoes, and clover when 15 tons of zeolite were added per hectare on Ukrainian sandy loam soils [35].

A comprehensive review by Cataldo et al. [36] supports these results, emphasizing that zeolite additions enhance soil fertility by improving nutrient retention and water holding capacity, resulting in elevated crop yields and reduced fertilizer leaching. The review also emphasizes the role of zeolites in mitigating environmental contamination by adsorbing heavy metals and ammonium ions, thereby limiting their uptake by plants and leaching into groundwater.

Animal Nutrition and Health. The addition of natural zeolite to the diet of poultry, cattle and pigs has certain benefits on feed intake, growth rate, weight gain, egg production and feed efficiency ratio [34]. In one of the earliest studies in Japan, the researchers used ≤10% mordenite and clinoptilolite as dietary supplements for poultry and swine. The results showed that test animals grow faster compared to control group, with a simultaneous decrease in the cost and amount of feed. Mature and young pigs fed diet containing 5% clinoptilolite gained about 16% more weight than animals receiving normal diets. The excrement of animals was less smelly due to the take up of NH_4_^+^ by natural zeolites and the severity and number of intestinal diseases reduced [3].

Additionally, the effect of dietary inclusion of zeolites in the diet of dairy cows on the yield of milk was studied, and the findings suggested that zeolites can be effectively utilized in the diet of dairy cows with a positive effect on the production of milk [37]. The efficacy of natural zeolites in safeguarding animals against mycotoxins has been well established. The observed capacity of natural zeolite to adsorb aflatoxins, which contaminate animal feed, has led to enhanced health in sheep, chickens, and pigs [3].

A study conducted by Abdelrahman et al. investigated the impact of integrating natural zeolite into broiler chicken diets. This study used 560 one-day-old female Ross-308 broilers, which were allocated into groups receiving diets with different zeolite concentrations (0, 5, 10, 15, and 20 g zeolite/kg) over a duration of 35 days. The findings demonstrated that zeolite supplementation, specifically at 10 g zeolite/kg, enhanced carcass features, meat quality attributes, and intestine metrics. Zeolite treatments significantly influenced litter pH, breast meat redness, cooking loss, chewiness, overall weight, and small intestine length. The study determined that zeolite, particularly in bigger particle sizes, serves as a desirable feed supplement in broiler diets, improving meat quality and intestinal health without negatively impacting growth performance, despite no significant changes in overall performance parameters [38].

Altogether, the studies reviewed suggest that natural zeolites play a vital role in enhancing both plant and animal agricultural systems. Their ability to improve soil fertility, water retention, and nutrient availability contributes significantly to increased crop yields and reduced environmental contamination. In animal husbandry, zeolites have shown considerable promise in improving feed efficiency, promoting growth and health, and mitigating the harmful effects of toxins in feed. As sustainable agriculture becomes increasingly important, the multifunctional benefits of natural zeolites establish them as valuable and environmentally friendly tools for improving productivity and health in agronomy, horticulture, and livestock production.

### 6.3. Aquaculture

According to the Food and Agriculture Organization of the United Nations (FAO), global aquaculture production reached an unprecedented 130.9 million tonnes in 2022, including 94.4 million tonnes of aquatic animals. This marked the first time in history that aquaculture surpassed capture fisheries, accounting for 51% of total aquatic animal production [39]. Zeolite is considered one of the most promising solutions that have emerged in recent years for achieving sustainability in the aquaculture industry. Natural zeolites have three vital positive impacts on aquaculture: (1) removal of ammonium ions from aquarium waters, hatcheries, and during transport; (2) production of oxygen-rich air for use in aeration systems in transportation and aquariums; and (3) enhancement of fish meals as a supplement. These attributes underscore the significance of natural zeolites in aquaculture [3].

Although the removal efficiency of zeolite for ammonium ions in salt water is less than in freshwater, the zeolite still has the highest efficiency and lowest cost compared with other materials such as activated carbon [40]. Zeolites have the ability to reduce the content of total dissolved solids (TDS) and suspended organic matter in fish ponds, which surely affects the quality of water, the health of fish and growth performance. In a field experiment carried out in Egypt, it was found that earthen ponds that were treated with zeolite resulted in a reduction in total dissolved solids (TDS) from 635 to 400 (mg/L), ammonium from 0.51 to 0.01 ppm and pH from 8.65 to 8.4 compared with ponds that were not treated with zeolite. Even with the addition of zeolite in the feed of fish, it was found that the turbidity of water is diminished [41].

A study by Hasan et al. investigated the impacts of zeolite on water quality, growth performance, heavy metal content, and health condition of farmed tilapia (*Oreochromis niloticus*). In this study, zeolite was applied directly into the water of treatment ponds at a rate of 200 g per decimal during pond preparation and 100 g per decimal every 15 days during the culture period. The results indicated that zeolite had a significant effect (*p* < 0.05) on all growth indices assessed across three experimental stages (0–42 days, 43–84 days, 85–126 days) in treatment ponds compared to control ponds. Notably, weight gain, specific growth rate, and daily growth coefficient were significantly improved in the zeolite-treated groups. Furthermore, the study observed a significant reduction in feed conversion ratio and improvements in water quality parameters, including decreased levels of ammonia and heavy metals. These findings suggest that zeolite application in aquaculture ponds can enhance fish growth performance and water quality, contributing to more sustainable aquaculture practices [42].

Taken together, the reviewed studies indicate that the growing dominance of aquaculture in global fish production highlights the urgent need for sustainable practices in the sector. Natural zeolites have demonstrated substantial promise in enhancing water quality, reducing environmental pollutants, and enhancing fish growth and health. Their cost-effectiveness, multifunctionality, and proven efficacy especially in recent studies support their continued application as a practical and sustainable solution in modern aquaculture systems.

### 6.4. Gas Processing

Gas processing represents one of the most important industrial applications of zeolites. Their well-defined microporous frameworks and high adsorption selectivity enable them to be widely used in gas dehydration, separation, and purification processes. In natural gas dehydration, zeolites efficiently adsorb water molecules, preventing corrosion and hydrate formation in pipelines and processing equipment. This application has made zeolite-based adsorbents a standard choice in the natural gas industry [7,12].

Another significant use is in air separation, particularly in the large-scale production of high-purity oxygen and nitrogen. Pressure swing adsorption (PSA) units employ zeolites—such as 5A and 13X—to selectively adsorb nitrogen, enabling continuous oxygen enrichment [7]. Zeolites are also applied in gas purification systems, where they effectively remove contaminants such as carbon dioxide, hydrogen sulfide, and other trace impurities from natural gas and industrial gas streams [12].

These examples underscore the versatility and efficiency of zeolites in gas processing, highlighting their critical role in ensuring energy efficiency, industrial safety, and the reliable supply of purified gases across multiple sectors, including energy production, metallurgy, and healthcare.

### 6.5. Construction

Concrete is the most common composite material that is used in the construction industry because of its excellent physical and mechanical properties. The usage of natural zeolites in concrete production has been preferred to obtain a good performance along with upgrading in terms of durability, structure, and mechanical properties [43]. Worldwide, the building and construction industry is considered to be the major consumer of natural zeolites [44]. The volcanic material is called phonolite, contains 45% zeolite, and is used in Germany, France, and Switzerland in the concrete industry [45]. In China, the production of cement is the industry with the largest zeolite consumption [46]. Natural zeolites that are utilized for building and construction materials have some unique characteristics such as accelerating admixture, non-alkali antifreeze, antibacterial agent, humidity-conditioning material, and carrier fluidizing agent for the workability of concrete [47].

The usage of natural zeolite in construction as a pozzolanic material dates back 3000 years to the time of the Greeks and Romans [48]. The calcium hydroxide, Ca(OH)_2_, formation during the hydration process of cement can contribute to the formation of extremely vulnerable concrete. This problem can be alleviated by adding natural zeolite to cement. The addition of natural zeolite to cement will form a dense microstructure of hardened concrete and cement since Al_2_O_3_ and SiO_2_ in natural zeolite form aluminate hydrate gels and calcium silicate hydrate [43]. Moreover, the addition of natural zeolite can reduce the penetration of chloride ions into concrete [49]. The addition of natural zeolite to concrete can also decrease the weight of concrete while improving its thermal insulation ability. All the results of research in construction showed that concrete or cement with suitable amount of natural zeolite has high performance ability [43,47].

Recent investigations have confirmed the effectiveness of natural zeolites in concrete applications. A recent study investigated the effects of incorporating zeolite powder at varying percentages (0%, 7.5%, and 10%) into concrete made with sea sand and seawater, using both ordinary Portland cement (OPC) and Portland Pozzolan Cement (PPC). The findings indicated that OPC-based concrete generally achieved higher compressive strength compared to PPC. Specifically, the addition of 7.5% zeolite to OPC yielded optimal compressive strength at 56 days. In terms of durability, PPC concrete with 10% zeolite demonstrated significant improvements, particularly in resistance to sulfate attack after immersion in a 5% Na_2_SO_4_ solution for up to 90 days [50]. Furthermore, research in 2024 highlighted that a 30% replacement of cement with zeolite powder in high-performance concrete (HPC) achieved a compressive strength of 85 MPa, while reducing the carbon footprint to approximately 659.72 kg CO_2_/m^3^. These findings demonstrate the ability of natural zeolites to enhance mechanical properties, durability, and sustainability in concrete applications [51].

Taken together, the reviewed literature demonstrates that natural zeolites offer considerable advantages as partial cement replacements in concrete production. Their pozzolanic activity, coupled with their unique physicochemical properties, contributes to enhanced compressive strength, improved durability against chemical attacks (e.g., sulfates and chlorides), and better thermal insulation. Furthermore, recent studies emphasize their role in promoting sustainable construction practices by enabling partial cement replacement, thus reducing CO_2_ emissions and overall environmental impact. Optimizing zeolite content in various cement formulations, including ordinary Portland cement and Portland pozzolan cement, has shown promising results in both mechanical performance and long-term durability. As the construction industry moves toward more sustainable and high-performance materials, natural zeolites emerge as a cost-effective and environmentally responsible solution with wide-ranging applications in modern cementitious systems.

### 6.6. Medicine and Biotechnology

Natural zeolites have shown significant promise in various medical and biotechnological applications, primarily due to their ion-exchange capacity and adsorption properties. These characteristics make them suitable for a range of functions, including the detoxification of human and animal organisms, separation of various cells and biomolecules, detection of biomarkers of different diseases, construction of biosensors, improvement of the immunity of animals, controlled gene and drug delivery and scavenging of oxygen. Furthermore, zeolites are promising as components of scaffold materials for bone, skin and vascular tissue engineering, and for bioactive and anticorrosive coatings for the implantation of bone [52].

Phillipsite, a kind of natural zeolite, has been shown to be a very efficient filtering material for eliminating ammonia ions from the dialysis solution used in the treatment of renal patients. This allows the purified solution to be reused several times in portable and home dialysis systems [3]. Zeolites have been utilized in human medicine as an anti-diarrheal remedy [53]. It has been reported that the healing time of surgical incisions and skin wounds can be decreased by using the powder of zeolite, thus making it a valuable therapy for athletes’ feet. Moreover, the anecdotal evidence obtained from the 3 mining sites in the USA suggests that the dust of zeolite at the mining site contributed to the faster healing of the scrapes and cuts of the workers. It is also stated that in Cuba, the powder of clinoptilolite is sprinkled on the cuts of cows and horses to speed the healing process [54].

Recent advancements have highlighted the biomedical potential of zeolite-based materials in wound care. Qi et al. [55] developed a thermoreversible hydrogel incorporating antibacterial zeolite nanoparticles, which exhibited a significant improvement in diabetic wound healing. The hydrogel exerted its therapeutic effects by scavenging reactive oxygen species (ROS), neutralizing pro-inflammatory cytokines, and promoting angiogenesis at the wound site. In a complementary study, El-Kasaby et al. [56] reported that purified clinoptilolite-tuff efficiently adsorbed malodorous amines from chronic wound exudates, indicating its potential for use in odor-controlling wound dressings. These findings highlight the significance of clinical-grade natural zeolites as multifunctional agents in advanced wound care, offering both healing and hygienic advantages.

Overall, zeolites exhibit considerable potential in medicine and biotechnology, owing to their ion-exchange properties, adsorption efficiency, and biocompatibility. Their applications span from detoxification and gastrointestinal therapies to advanced wound care and tissue regeneration. Recent innovations, including zeolite-based hydrogels and odor-controlling dressings, further support their relevance in addressing complex clinical challenges. As research continues to explore emerging biomedical applications, zeolites are poised to become integral components in the development of safe, cost-effective, and multifunctional therapeutic solutions.

### 6.7. Radioactive Waste Remediation

Natural zeolites first attracted significant attention for their usage in radioactive waste management in the 1950s [57]. Their initial application was primarily focused on the management of low-level radioactive waste. Recent advancements have broadened their application to include the treatment of intermediate and high-level radioactive waste [58]. Natural zeolites provide several beneficial attributes, including: (i) affordability and extensive availability, (ii) high selectivity for certain ions, (iii) significant ion exchange capacity, and (iv) remarkable radiological stability under beta, alpha, and gamma radiation. Due to these properties, natural zeolites are now among the most widely used adsorbents in the remediation of radioactive wastewater [12,58].

Numerous reports have confirmed the superior performance of different natural zeolites, particularly clinoptilolite, in capturing a wide range of radionuclides, including cesium (Cs) and strontium (Sr). Natural zeolite (clinoptilolite) has been widely utilized at a large scale for the removal of cesium-137 (Cs-137) from low-, intermediate-, and high-level radioactive wastewaters. Following the Three Mile Island nuclear accident in Pennsylvania, both natural zeolite (chabazite) and synthetic zeolite (LTA) were evaluated for their effectiveness in removing Cs-137 and strontium-90 (Sr-90) from contaminated water. In the United Kingdom, clinoptilolite has been used for the treatment of low-level radioactive effluents at the Sellafield nuclear power facility, targeting the removal of Cs-137 and Sr-90. Additionally, natural clinoptilolite and mordenite have been utilized to decontaminate radioactive effluents that were discharged from nuclear power plants in the Chernobyl region [59].

Moreover, the adsorption of strontium and cesium utilizing Iranian natural zeolite was investigated. Based on the obtained results, the removal efficiency for cesium was around 67.8%, whereas for strontium it was roughly 93.5%. [60]. Mordenite, clinoptilolite, chabazite and synthetic mordenite were examined for their efficiency in removing cesium from low-level radioactive liquid waste; the gained results showed that the natural chabazite has the highest capacity towards cesium compared with the other zeolite materials [61].

Beyond cesium and strontium, natural zeolites, along with their modified and synthetic counterparts, have also been employed for the removal of various other radioisotopes, including cobalt, uranium, and thorium. The adsorption capacity of natural clinoptilolite for cobalt ions in aqueous solutions has been extensively investigated, with results confirming its high efficiency as an adsorbent [62]. In another study, natural Greek zeolite and bentonite were evaluated for uranium removal from liquid solutions, where the zeolite exhibited superior performance [63]. Additionally, the adsorption behavior of uranium and thorium using Jordanian zeolitic tuff—primarily composed of phillipsite and chabazite—was examined, and findings suggested that this material is highly effective for removing these radionuclides from acidic solutions [64]. More recently, phosphate-modified clinoptilolite has demonstrated excellent performance in removing thorium (IV) from aqueous solutions, confirming its potential as a robust and efficient adsorbent for radioactive actinide remediation [65].

In summary, natural zeolites—particularly clinoptilolite, chabazite, and mordenite—have proven to be highly effective materials for the remediation of radioactive waste due to their ion exchange capacity, selectivity, stability under radiation, and cost-effectiveness. Their successful application in various nuclear incidents and industrial-scale treatments underscores their value in removing key radionuclides such as cesium-137, strontium-90, cobalt, uranium, and thorium. Advances in modification techniques, such as phosphate functionalization, have further enhanced their adsorption capabilities, especially for actinides like thorium. These findings collectively highlight the vital role of natural and modified zeolites in current and future strategies for radioactive waste management, particularly in supporting sustainable and safe environmental remediation practices.

### 6.8. The Current Challenges and the Future Applications of Zeolite

Nowadays, the world is facing many serious threats. For instance, (i) lack of freshwater and drought caused by climate change, (ii) dangerous pandemics such as the COVID-19 pandemic, and (iii) economic and political instabilities, which resulted in a slowing down of economic growth, development, and scientific research, and even raised questions about the survival of human beings.

With the current usage of zeolite in water processing, animal husbandry, agriculture, numerous industries (construction, chemical, aquaculture, etc.), medicine, biotechnology, etc., the future of zeolite applications opens up new opportunities for research, improvement, and technological applications in still undiscovered fields such as antimicrobial and medication applications [66], which will allow new worldwide markets for zeolite.

Additionally, the zeolite application in technologies that could contribute to the decrease of carbon dioxide emissions, which are the primary driver of global climate change, could have an important role in its uses in the near future [13]. Recent investigations have provided further support for the role of zeolites in addressing climate-related technological needs. A 2020 experimental study demonstrated that Na^+^- and Ca^2+^-exchanged clinoptilolite exhibited approximately 25% higher CO_2_ uptake than commercial zeolite 13X at 338 K under dynamic conditions [67]. A 2024 review by Grifasi et al. further supports these findings, noting the role of clinoptilolite’s adjustable cation composition in enhancing CO_2_ adsorption, particularly at elevated temperatures [68]. These findings highlight the viability of clinoptilolite as a low-cost and efficient adsorbent for carbon capture technologies under real-world flue gas conditions.

Looking ahead, future research should prioritize improving zeolite selectivity, optimizing regeneration protocols, and advancing scalable production methods for industrial use. Continued innovation in their use as carbon adsorbents, antimicrobial agents, and biomedical materials underscores the increasing relevance of zeolites in addressing both environmental and public health challenges.

Recent years have witnessed remarkable progress in the design and application of zeolite membranes, which represent a promising frontier for future technologies. Zeolite membranes combine the unique molecular sieving properties of zeolites with the mechanical stability of membrane systems, enabling highly selective and energy-efficient separation processes. They have been widely investigated for gas separation (e.g., CO_2_/CH_4_, N_2_/O_2_), pervaporation (e.g., dehydration of alcohols and organics), and water purification, where they demonstrate superior selectivity and durability compared to conventional polymeric membranes [12,13].

Particularly, zeolite membranes hold great potential in addressing global energy and environmental challenges by providing low-energy alternatives to traditional separation methods. Their tunable pore size and hydrophilic/hydrophobic properties allow precise tailoring for targeted separations, making them attractive for industrial-scale applications in chemical processing, clean energy production, and environmental remediation. As ongoing research continues to improve their fabrication methods and long-term stability, zeolite membranes are expected to play a pivotal role in the future landscape of zeolite applications.

## 7. Commercialization of Zeolites

Zeolites have achieved remarkable commercial success in several industrial sectors. In the petrochemical industry, zeolite-based catalysts such as Y-zeolite and ZSM-5 are indispensable in fluid catalytic cracking (FCC), hydrocracking, and isomerization processes, significantly improving fuel yield and selectivity1960s [7,12]. In gas processing and separation, zeolites 5A and 13X are widely applied in pressure swing adsorption (PSA) systems for the production of oxygen and nitrogen, and for the purification of natural gas by removing CO_2_ and H_2_S [12]. Zeolite A has also been successfully commercialized as a phosphate substitute in detergents, providing environmentally friendly cleaning performance. Additionally, natural zeolites (clinoptilolite, chabazite, mordenite) are extensively used in water treatment and environmental remediation, where their ion-exchange capacity allows effective removal of heavy metals, radionuclides, and ammonium [13].

Despite these successes, the commercialization of zeolites in other sectors, such as medicine, biotechnology, and advanced membranes, is still in its early stages. This is largely due to several limiting factors, including high synthesis costs, challenges in scalability, and concerns about long-term stability under operational conditions. Furthermore, broader adoption is constrained by diffusion limitations in purely microporous frameworks, regulatory hurdles in biomedical applications, and competition from alternative porous materials such as activated carbons, mesoporous silicas, and metal–organic frameworks (MOFs) [7,12,13,23].

A well-argued examination of these factors highlights the critical issues limiting the broader commercialization of zeolites. Economic feasibility remains a barrier, as advanced zeolite synthesis often requires expensive structure-directing agents and energy-intensive processes. Scalability is another challenge, since only a limited number of zeolite frameworks (A, X, Y, ZSM-5) are currently produced on a large scale, while newer types face obstacles in industrial manufacturing. Framework stability under hydrothermal or chemically harsh conditions can restrict long-term use, particularly in catalysis and membrane applications. Moreover, the market presence of alternative porous materials—often cheaper or more tunable—further limits zeolite penetration in emerging sectors [12,13,23].

Addressing these limitations will require innovations in green and cost-effective synthesis routes, the design of hierarchical or composite frameworks to overcome diffusion barriers, and the development of durable zeolite-based membranes and composites. Such advances could significantly expand the industrial footprint of zeolites beyond their already established markets, unlocking new opportunities in clean energy, advanced catalysis, environmental remediation, and biomedicine.

## 8. Conclusions

Natural zeolites are abundant, environmentally compatible materials with distinctive structural and ion-exchange properties that enable diverse applications in environmental remediation, agriculture, aquaculture, construction, medicine, and radioactive waste management. Clinoptilolite, chabazite, and mordenite remain the most widely utilized, with performance largely determined by geological origin and modification strategies. Recent advances—including tailored functionalization, biomedical integration, and carbon capture—demonstrate their adaptability to emerging global challenges. Nevertheless, improving selectivity, regeneration efficiency, scalability, and addressing commercialization barriers remain critical. Sustained interdisciplinary efforts will be crucial to translate the versatile properties of zeolites into scalable, cost-effective, and sustainable solutions, thereby ensuring their continued relevance in science, technology, and industry.

## Data Availability

No new data were created or analyzed in this study. Data sharing is not applicable to this article.
